# Data enhanced iterative few-sample learning algorithm-based inverse design of 2D programmable chiral metamaterials

**DOI:** 10.1515/nanoph-2022-0310

**Published:** 2022-09-06

**Authors:** Zeyu Zhao, Jie You, Jun Zhang, Shiyin Du, Zilong Tao, Yuhua Tang, Tian Jiang

**Affiliations:** State Key Laboratory of High Performance Computing, College of Computer, National University of Defense Technology, 410073, Changsha, China; Defense Innovation Institute, Academy of Military Sciences PLA China, 100071, Beijing, China; Beijing Institute for Advanced Study, National University of Defense Technology, 100020, Beijing, China

**Keywords:** deep learning, few-sample, inverse design, programmable metamaterial

## Abstract

A data enhanced iterative few-sample (DEIFS) algorithm is proposed to achieve the accurate and efficient inverse design of multi-shaped 2D chiral metamaterials. Specifically, three categories of 2D diffractive chiral structures with different geometrical parameters, including widths, separation spaces, bridge lengths, and gold lengths are studied utilising both the conventional rigorous coupled wave analysis (RCWA) approach and DEIFS algorithm, with the former approach assisting the training process for the latter. The DEIFS algorithm can be divided into two main stages, namely data enhancement and iterations. Firstly, some “pseudo data” are generated by a forward prediction network that can efficiently predict the circular dichroism (CD) response of 2D diffractive chiral metamaterials to reinforce the dataset after necessary denoising. Then, the algorithm uses the CD spectra and the predictions of parameters with smaller errors iteratively to achieve accurate values of the remaining parameters. Meanwhile, according to the impact of geometric parameters on the chiroptical response, a new functionality is added to interpret the experimental results of DEIFS algorithm from the perspective of data, improving the interpretability of the DEIFS. In this way, the DEIFS algorithm replaces the time-consuming iterative optimization process with a faster and simpler approach that achieves accurate inverse design with dataset whose amount is at least one to two orders of magnitude less than most previous deep learning methods, reducing the dependence on simulated spectra. Furthermore, the fast inverse design of multiple shaped metamaterials allows for different light manipulation, demonstrating excellent potentials in applications of optical coding and information processing. This work belongs to one of the first attempts to thoroughly characterize the flexibility, interpretability, and generalization ability of DEIFS algorithm in studying various chiroptical effects in metamaterials and accelerating the inverse design of hypersensitive photonic devices.

## 1 Introduction

Tremendous literature has grown up around the theme of chiral metamaterials, which not only provides new insights into light–matter coupling effects at the nanoscale and strong chiroptical interactions, but also demonstrates dramatic application prospect in chiral sensing and filtering, polarization-selective communication, biological detection, and chirality-relevant quantum optics [1–4]. Significantly, optical chirality, a ubiquitous phenomenon in universe, has played a pivotal role in the development of life science [5], biochemistry [6], pharmaceutics [7], spectroscopy [8], and quantum computing [9]. Circular dichroism (CD) effect, serving as one prominent category of optical chirality, identifies the absorption difference of metamaterials under irradiation of the right- (RCP) and left-circularly polarized (LCP) light excitation [10, 11]. In particular, the CD effect is normally decided by either the handedness of chiral metamaterials or their chiral parameters [12], making possible for the engineering of chiroptical response with a huge degree of flexibility, which is totally different from natural molecules that own weak chirality and are inconvenient to change the size [13]. One distinct and distinguished chiral metamaterials is the two-dimensional (2D) version, which demonstrates significant advantages in small optical losses, compact dispersion, simple fabrication process, and excellent reconfigurability [14–16]. Further to that, 2D diffractive chiral metamaterials have established as alluring and attractive platforms to study optical chirality and the relevant but unrevealed mechanisms, in which much larger CD response is observed at higher-order diffracted beams than the zeroth-order [17]. Though there has been a moderate increase in the investigations of diffractive metamaterials possessing divergent geometries [18–20], a fast, intelligent and vigorous tool is still needed to perform inverse design for such metamaterials, considering their widespread applications in ultrafast detection, nonlinear chiroptical phenomena, and hyper-sensitive ultrathin devices.

Recently, deep learning (DL) algorithm, an important branch of machine learning (ML), has been applied as an attractive and intriguing method in scientific studies owing to the rapid progress of computer technology. The utilization of DL covers tremendous aspects, such as medicine [21–23], finance [24–26], and nature language processing [27–29]. In particular, the last five years have witnessed a great development in the field of nanophotonics thanks to DL approach, which facilitates the solution of many nonlinear and nonintuitive problems, with the inverse design of tremendous devices included [30–33]. Though traditional numerical simulation methods (e.g. FDTD, FEM, RCWA) are often used to characterize the optical response of nano-devices at the cost of expensive computing time and resources, they cannot provide explicit design principles, neither. The DL network is essentially a statistical learning method, which makes reasoning and prediction through the patterns and connections reflected by data, seeking to accomplish inverse design in a fast and efficient way, rather than focusing on the design mechanisms. This makes possible for the on-demand inverse design of a variety of 2D programmable chiral metamaterials in a highly-accurate and ultrafast manner. In 2018, Peurifpy et al. [34] applied neural networks with 50,000 samples to assist the inverse design of a multilayer dielectric spherical nanoparticle, which was a milestone in the domain. Liu et al. [35] designed a thin film composing of alternating layers of SiO_2_ and Si_3_N_4_ through employing a tandem neural network and 500,000 instances to make the results converge better. Furthermore, Liu et al. [36] made a successful attempt at designing metasurfaces in 2018, which revealed the potential of generative adversarial networks (GANs) in the design of such structures. In 2021, Kong et al. [37] proposed a bidirectional cascaded deep neural network with a pretrained autoencoder for rapid design of dielectric metasurfaces in the range of 450–850 nm, replacing the traditional time-consuming and laborious design methods. Raju et al. [38] fabricated the algorithm-designed plasmonic patterns on the prepared nanolaminate in 2022, showing the practicability of deep learning. All these works suggest that DL algorithm has demonstrated obvious advantages in speed, flexibility, and accuracy. In turn, research groups can complete the design with similar effect in much less time compared with traditional methods.

However, traditional DL algorithm has two drawbacks. Firstly, the training process is especially data-consuming. In addition to the aforementioned researches, there are many works troubled by this problem. For example, in 2021 Sun et al. [39] trained an improved K-nearest neighbor algorithm to design meta-atoms using 2.7 × 10^7^ sets of spectra, taking approximately 240 h to yield the data. Zhang et al. [40] employed 70,000 training coding patterns to train the machine learning algorithm, in order to realize intelligent inverse design of metasurfaces in 2019. Ma et al. [41] collected 30,000 samples to complete on-demand design of chiral metamaterials in 2018. Coincidentally, the cost of data acquisition is unimaginable, which makes DL algorithms difficult to be popularized. Secondly, the architecture is too monolithic to be flexible. Many research efforts lack task decomposition. As tasks become more complex, the size of the network grows which increases the difficulty of training and degrades the final effect.

**Figure 1:**
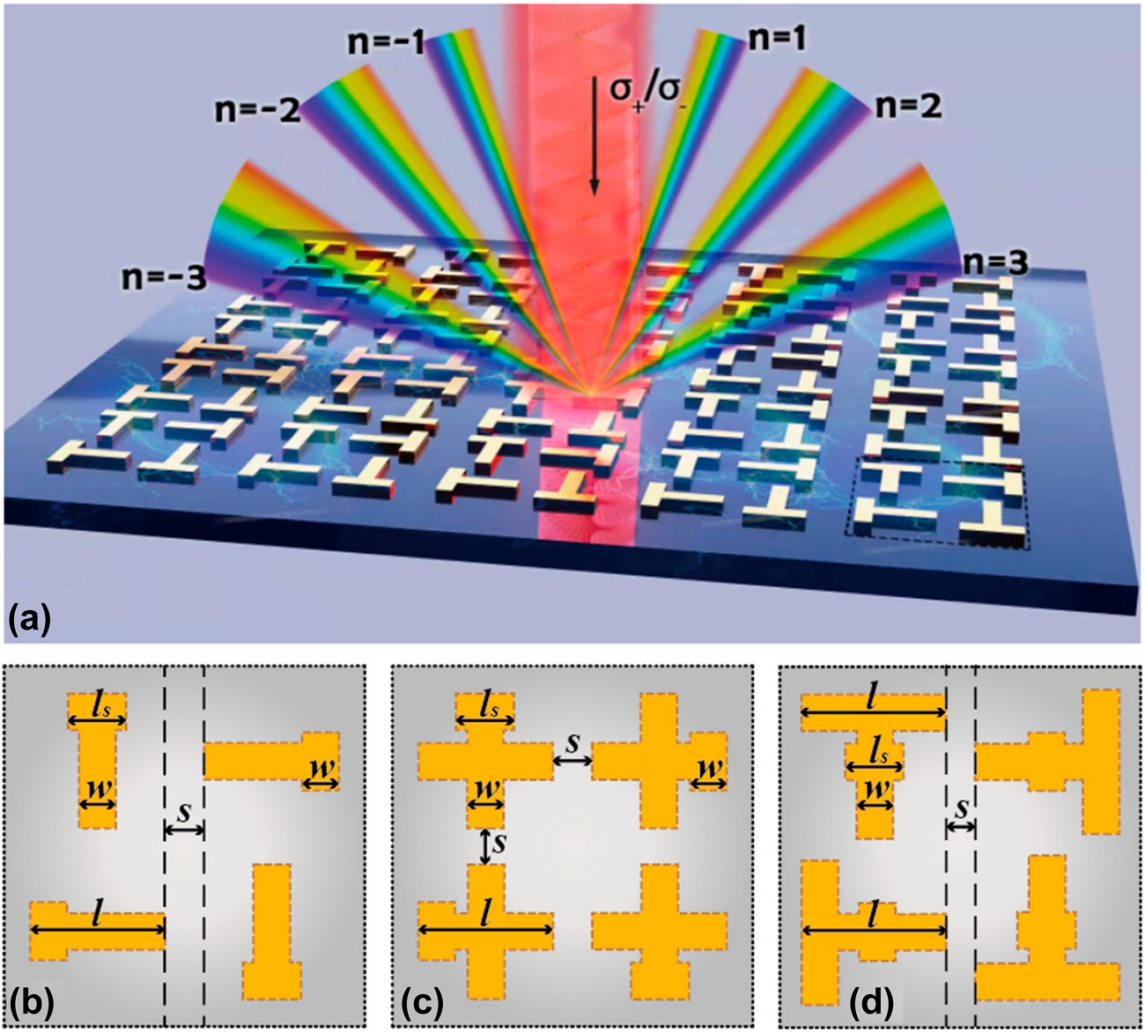
Schematic of different chiral metamaterials. (a) Schematic of the higher-order diffraction patterns when the circularly polarized light irradiates the S1 metamaterial. (b)–(d) Schematics of the metallic array’s unit cell for S1, S2, and S3, respectively.

**Figure 2:**
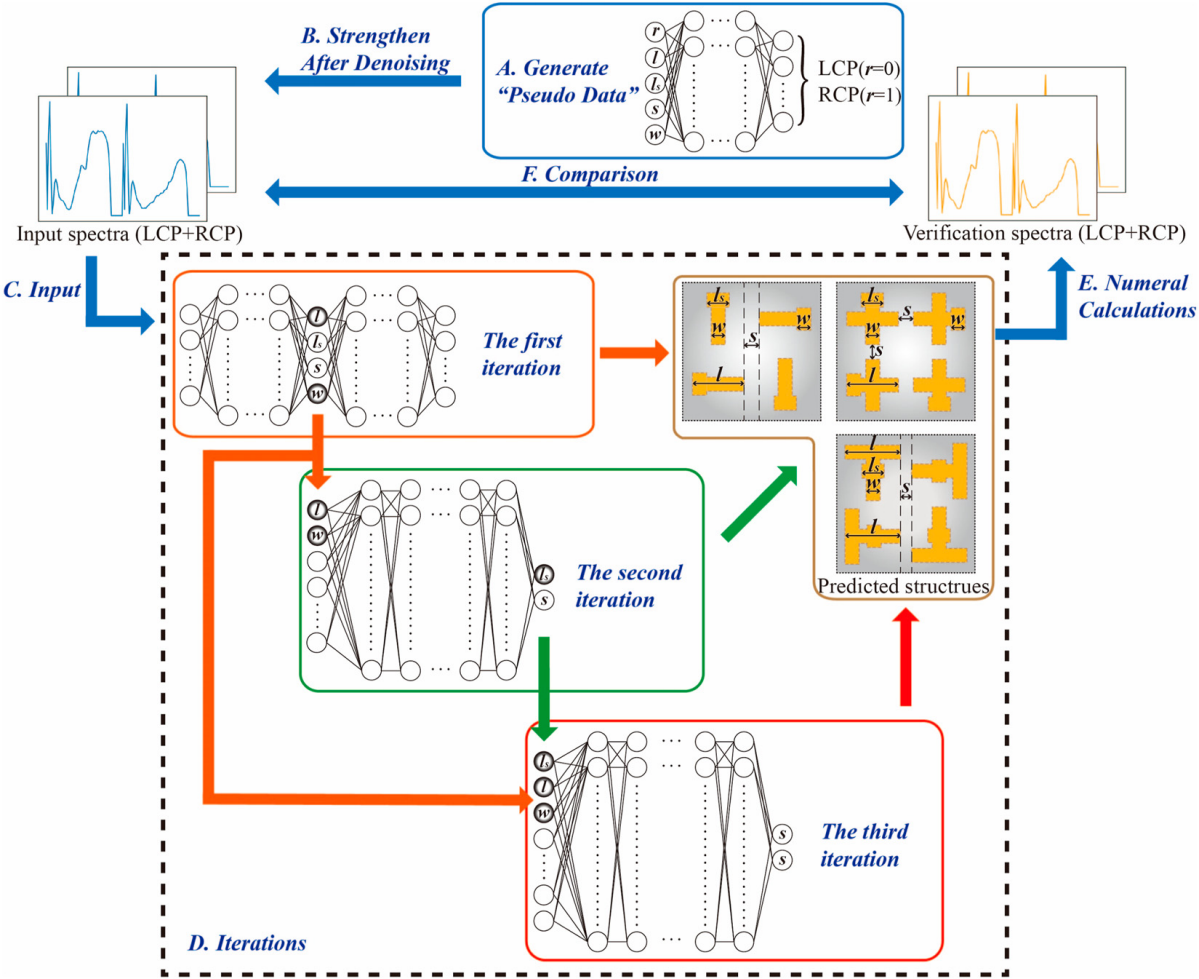
Schematics of the DEIFS algorithm for inverse design of 2D chiral metamaterials. The algorithm can be divided into two main stages, data enhancement (blue solid box) and iterations (black dashed box). The first stage is using the results of the forward prediction network to strengthen the dataset ((A) and (B)). The second stage is utilizing the spectra to predict corresponding geometric parameters with the help of additional inputs namely parameters with smaller errors ((C) and (D)). Then, the verification spectra are compared with the input spectra to verify whether the results of the algorithm are satisfactory ((E) and (F)).

In this work, targeting at designing 2D chiral metamaterials efficiently, an algorithm called data enhanced iterative few-sample (DEIFS) has been introduced and applied. DEIFS has two core ideas. One is DATA AUGMENTATION, aimed at augmenting the original dataset making it possible to train large networks with small amounts of data. The other is REGRESSOR CHAINS [42], using multiple small regressors instead of a monolithic network to iteratively solve the problem in a chain, taking advantage of the potential conditional dependence between data and improving algorithm performance. In particular, DEIFS is used to solve the inverse design problem of chiral structures with a four-dimensional parameter space, shown as Figure 1. By means of DEIFS network, we study the chiroptical responses of variously-shaped 2D chiral metamaterials with different gold lengths, widths, bridge lengths, and separation spaces. The process of DEIFS can be divided into two stages. One is data enhancement, focusing on dataset generation. The other is iterations, aiming at solver building. Specifically, in stage one, “pseudo data” is generated by exploiting a forward prediction network which can predict the optical response of chiral samples with various geometric parameters, as supplements of the original training data so that the network can be well trained after denoising. Notably, “original training data” refers to the data generated by the traditional rigorous coupled wave analysis (RCWA) method, which should be distinguished from the aforementioned “pseudo data”. In stage two, the inverse design is performed by a tandem network composed with a forward network and an inverse network. Additionally, parameters with fewer design errors are picked out as additional inputs for the next round of inverse design to take full advantage of the potential relationships existing between the geometric specifications. Generally, the inverse design that satisfies the spectroscopic demands can be realized after two or three iterations. Importantly, the DEIFS algorithm is confirmed to be a strong and promising tool that can realize efficient inverse design for multiple 2D diffractive chiral metamaterials in a highly-accurate (about 96%) and ultrafast manner (0.75 s for 737 testing samples), which is obviously superior to traditional inverse design methods and many machine learning methods in terms of flexibility, scalability, and time consumption. The entire designing algorithm and dataflow are schematically presented in Figure 2.

## 2 Design of 2D chiral metamaterials with different structures

The crucial theme is to design the demanded structures of 2D chiral metamaterial effectively given less third-order diffracted circular dichroism response data. The higher-order diffraction patterns when the T-like metamaterial is irradiated by the circularly polarized light are shown as Figure 1(a). Moreover, Figure 1(b)–(d) distinctly exhibit the basic cells of three chiral metamaterials, denoted as S1, S2, and S3, in order of structure from simple to complex. Their depth profiles are shared: a Si layer acts as the substrate, followed by a 200 nm SiO_2_ layer and a 10 nm Cr film stacked on the substrate sequentially. The uppermost layer is fabricated as 30 nm-thick gold arrays. Besides investigating multiple structures, different geometric parameters are considered, including the gold length (*l*) (0.8–2.0 μm), width (*w*) (0.1–0.3*l*), separation space (*s*) (0.1*l*–*l*), and bridge length (*l*_*s*_) (0.4*l*–*l*). These parameters are not on a uniform scale, which can bring inconvenience to feature extraction and learning of the algorithm, ultimately leading to terrible design results. Nevertheless, the variations of the graphic parameters can be considered discrete. The preparation precision of a device must not only be greater than or equal to a minimum resolution, but the adjustment of parameters must also be an integer multiple of this precision. What’s more, considering the balance between density and efficiency in the data generation process, parameters must be discretized. Therefore, geometric parameters can be normalized to the same nonnegative scale in a less complicated way. Assign a precision to each parameter, thus the normalization method can be represented as:

(1) $$x_{\text{nor}}=\frac{x-x_{\min}}{\varepsilon }$$

where *x* means the actual value, *x*_min_ means the minimum value and *ε* denotes the precision. As parameters vary in nonunique ranges, appropriate *x*_min_ and *ε* are necessary to be chosen to guarantee that the labels of several tasks are on a unified scale. The minimum value of each geometric parameter in the dataset is regulated to be zero so that other values can be positive integers. Furthermore, since the parameters are normalized to integers, the prediction of them should also be rounded and truncated to acquire more exact results. In other words, those errors less than 0.5 are shrunken to 0, and those greater than 0.5 are generally enlarged to 1. It requires that the prediction errors should be as small as possible, otherwise rounding and truncation will in turn magnify them.

To generate original training data, the rigorous coupled wave analysis (RCWA) method is employed. With its assistance, we obtain a dataset containing 7358 pairs of the LCP and RCP spectra whose wavelength range from 0.2 to 1.775 μm, for the three structures in different gold lengths, widths, separation spaces, and bridge lengths. The dataset is divided into three parts: 70, 20, and 10% for training, validation, and testing, respectively.

## 3 Data enhanced iterative few-sample algorithm

The working process of DEIFS algorithm is shown in Figure 2, innovating on data source and additional inputs. Two key and essential parts of DEIFS algorithm can be immediately found from Figure 2, namely data enhancement and iterations. Regarding data enhancement, this algorithm works mainly due to the difference in the mathematical principles behind forward prediction and inverse design. Precisely, the forward prediction is essentially a process of fitting and solving Maxwell’s equations, while the inverse design is a more difficult and data-dependent process of finding the inverse of Maxwell’s equations, even in cases where only pseudo-inverses exist. Importantly, this demand for data can be quickly satisfied through data enhancement. With regard to iterations, its application is primarily according to the network scale that is proportional to the problem complexity. Furthermore, the spectrum is deterministic in inverse design, which imposes conditional dependence on geometrical parameters that are otherwise independent. Therefore, applying an iterative method, regressor chains, which can take advantage of the dependencies and accomplish the inverse design task easily and flexibly is a wise choice.

**Figure 3:**
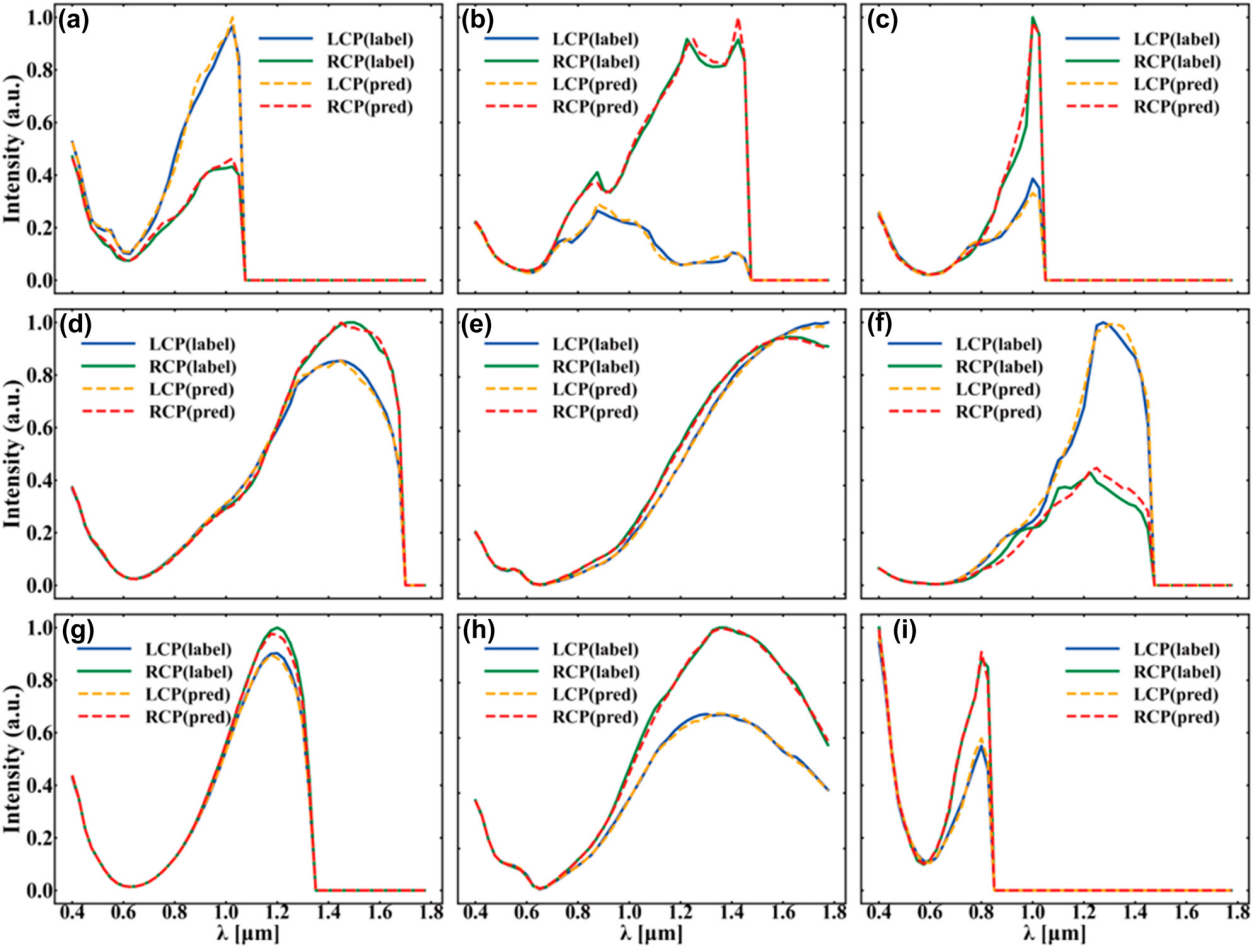
Comparison of the third-order diffracted chiroptical response calculated by RCWA (label, solid lines) and the forward prediction network (pred, dotted lines). (a)–(i) The LCP (blue and yellow) and RCP (green and red) spectra for S1, S2, and S3 from top to bottom respectively. Specially, the values of the geometric parameters are widely distributed to highlight the effect of the forward prediction network, which are shown in Table 1.

The exhaustive process of DEIFS is as follows: Firstly, a fully connected forward prediction network with seven hidden layers is trained with the original training dataset. The input of the network are four geometric parameters and the polarization direction, and the output of the network is an LCP or RCP spectrum with 64 data points indicating wavelength from 0.2 to 1.775 μm. Figure 3 shows the performance of the network, the basis of data augmentation. The values of geometric parameters corresponding to spectra in Figure 3 are shown in Table 1. Note that the absolute values of original spectra are extremely small, usually varying between 10^−3^ and 10^−7^. To make feature extraction easier and learning outcomes better, it is necessary and worthwhile to transform the spectra to a dimension with greater absolute values in the following manner:

(2) $$y\prime =-\mathrm{lg}\left(y+10^{-11}\right)$$

where *y* represents the spectra before processing and *y*′ denotes the spectra after computing. Here, 10^−11^ avoids the influence of zero values on the pretreatment commendably and minimizes its impact on the ultimate outcome concurrently. The loss function of the forward network is the mean absolute error (MAE), which can be calculated in the following way:

(3) $$Loss_{\mathrm{forward}}=\frac{1}{n}\overset{n}{\underset{i=1}{\sum }}abs\left(y_{\mathrm{pred}}^{\prime i}-y_{\mathrm{label}}^{\prime i}\right)$$

where *n* is the batch size, $y_{\mathrm{pred}}^{\prime }$ represents the prediction of the spectra, and $y_{\mathrm{label}}^{\prime }$ stands for the corresponding true optical spectra. The error of the network results is in the 10^−2^ order of magnitude. In this case, the predicted spectra are reliable because the converted value of a data point is mostly a positive number greater than three and less than eleven. Therefore, the dataset can be intensified using the “pseudo data” created by the optical responses of adjusted novel parameter combinations, which are not generated via RCWA simulations. By this means, 21,541 pairs of spectra are predicted within 0.68 s and supplemented to the dataset after necessary interpolation denoising. The “pseudo data” are added to the training and validation datasets in accordance with the proportion of 70 and 30%, respectively. To avoid the possible spectral error, “pseudo data” is not supplemented to the testing dataset. To some extent, this partition may bring the problem of inconsistent data distribution between testing dataset and training dataset. However, in the case that the spectral error of the “pseudo data” is insignificant, the distribution inconsistency causes minimal impact on the training.

**Table 1:** The values of geometric parameters corresponding to spectra in Figure 3.

Figure	Values of geometric parameters			
	*w*	*s*	*l*_*s*_	*l*
3(a)	0.125*l*	0.15*l*	0.45*l*	1.4 μm
3(b)	0.2*l*	0.3*l*	0.8*l*	1.7 μm
3(c)	0.3*l*	0.2*l*	0.6*l*	1.3 μm
3(d)	0.1*l*	0.5*l*	0.9*l*	1.7 μm
3(e)	0.175*l*	0.85*l*	0.55*l*	1.9 μm
3(f)	0.3*l*	0.7*l*	0.4*l*	1.3 μm
3(g)	0.1*l*	1.0*l*	0.5*l*	1.0 μm
3(h)	0.225*l*	0.95*l*	0.55*l*	1.5 μm
3(i)	0.3*l*	0.4*l*	0.6*l*	0.9 μm

The theme of the second stage is inverse design of 2D chiral structures; taking full advantage of the data including the entries produced from RCWA and artificial neural network approaches, whose process is called iteration. The objectives of iterations are mainly achieved in three steps. The first iteration requires a tandem network which is based on an inverse neural network and a forward network, both consisting of seven hidden layers. The purpose of the tandem network is to obtain precise geometric parameters. When the parameter prediction error occurs, the optical functional requirements should be guaranteed as far as possible. Notably, before the tandem network’s training process, the forward network needs to be trained first. The input of the forward network is four graphic parameters, while the output is a 128 bit vector consisting of the corresponding LCP and RCP. Predicting whether a spectrum belongs to LCP or RCP accurately is quite difficult for the inverse network, which is the reason why the network with 5 bit input and 64 bit output trained in the first stage is not employed here. The weights of the new trained forward network are copied to the tandem network and frozen, that is, these weights are not updated in subsequent training. In order to speed up the experimental process, the inverse design network is individually trained to find hyperparameters with better availability. It is less time-consuming than direct searching to obtain hyperparameters when training the tandem network. Moreover, this part of weights is not replicated in the tandem network. The found hyperparameters are only used to train the tandem network from scratch. The loss of the tandem network is denoted as following formula:

(4) $$\begin{array}{ll} Loss_{\text{tandem}} & =\alpha MAE\left(spec_{in},\;spec_{\mathrm{out}}\right)\\ & \;+\beta \left(MAE\left(wx_{\mathrm{true}},\;wx_{\mathrm{pred}}\right)\right.\\ & \;\left.+\gamma MAE\left({\mathrm{wx}}_{\mathrm{true}}^{\prime },\;{\mathrm{wx}}_{\mathrm{pred}}^{\prime }\right)\right) \end{array}$$

where *α* and *β* are the scale coefficients which are responsible for maintaining the balance between spectral error and parameter error, *spec*_*in*_ and *spec*_*out*_ represent the input and output of the tandem network respectively, *x*_*true*_, *x*_*pred*_, $x_{\mathrm{true}}^{\prime }$ and $x_{\mathrm{pred}}^{\prime }$ represent the true and predicted values of original training data and “pseudo data”, respectively, *γ* denotes a scale factor used to distinguish the two kinds of data because of the inevitable error of the “pseudo data” even after denoising, and *w* means a vector containing the different weights of the four parameters according to their influence on the spectra. The hyperparameter *w* can be computed in the following way:

(5) $$\begin{array}{ll} {diff}_{m} & =\frac{2}{n\left(n-1\right)}\sum_{i=1}^{n}\sum_{j=i+1}^{n}\frac{MAE\left(spec^{i},\;spec^{j}\right)}{abs\left(k_{m}^{i}-k_{m}^{j}\right)}\\ & \;\left(k_{m}^{i}\neq k_{m}^{j},m=w,s,l_{s},l\right) \end{array}$$

(6) $$w_{m}=\frac{diff_{m}}{\min\left\{diff_{w},diff_{s},diff_{l_{s}},diff_{l}\right\}}\;\;\left(m=w,s,l_{s},l\right)$$

where *n* represents the size of the training dataset divided from original training data, *m* means the four parameters and *k* means the value of the geometric parameters after normalization. Thus, the algorithm pays more attention to the more important parameters in spectrum, so as to better grasp the nonlinear relationship between geometric parameters and spectra. On the other way, a more exact prediction of the important parameters is conducive to reduce the overall spectral error. This method has been proved to be feasible and effective in another inverse design problem [43]. The tandem network can be used to preliminarily predict the structures. On this foundation, two parameters with better effect are chosen as additional inputs for the latter two steps. The criterion for selection is MAE of the parameter less than 0.05, so width and gold length are selected for the three structures. Half of the geometric parameters do not benefit from the iteration in designs to some extent. On the one hand, their errors (MAE < 0.05) are very low compared to their normalized minimum variation (i.e., 1), leaving little space for improvement. On the other hand, providing information gain at the cost of tolerating small errors facilitates better prediction of other parameters.

The next two steps are largely identical with only minor differences. Their networks are both with seven hidden layers. Their inputs are vectors embodying spectra and additional parameter predictions, and their outputs are the other geometric parameters. Their loss functions are similar in every detail which can be represented as:

(7) $$Loss_{\mathrm{two}}=MAE\left(x_{\mathrm{true}},x_{\mathrm{pred}}\right)+MAE\left(\gamma x_{\mathrm{true}}^{\prime },\gamma x_{\mathrm{pred}}^{\prime }\right)$$

where the meaning of each variable in this formula is the same as formula (4). The third parameter is elected in the second iteration. It is the bridge length for S1 and S2 and separation space for S3. Significantly, due to the operational characteristics of the code, the output of the third iteration is the reuse of the fourth parameter to ensure the correct outcomes. After these three iterations, a prediction of the four graphic parameters can be generated by correlating the preceding iteration results.

After getting the precise predictions of the parameters, the corresponding optical responses can be acquired and compared with the true value to verify the effectiveness of the algorithm. In this comparison, through satisfying the functional requirements for spectra successfully, the superiority of the algorithm can be demonstrated.

The relevant neural network in the algorithm is coded using TensorFlow2-gpu, an open-source artificial intelligent framework. The Adam optimization algorithm is selected as the optimizer to train the network. As for searching the hyperparameters, an open-source automated machine learning toolkit named NNI developed by Microsoft is chosen to make the experimental process faster and more efficient.

## 4 Inverse design of the chiral metamaterials

In this section, intending to reveal the advantages of the DEIFS algorithm, it is applied to realize the accurate and efficient inverse design of three 2D chiral structures. The testing dataset for each type of metamaterial is the 737 randomly selected samples from the original training data which contains 7358 items. The inputs of the DEIFS algorithm are 128-data-point vectors containing LCP and RCP, and the outputs are 4 bit vectors denoting the geometric parameters. The priority target is to get accurate predictions of the four structure parameters, and the algorithm should complete the optical target at least. Therefore, the mean absolute percentage error (MAPE) not greater than 5% in the spectrum corresponding to the predicted parameters relative to the spectrum of the true parameters is set as the standard. The MAPE can be denoted clearly using the following equation:

(8) $$MAPE=\frac{1}{n}\overset{n}{\underset{i=1}{\sum }}abs\left(\frac{y_{\mathrm{pred}}^{\prime }-y_{\mathrm{label}}^{\prime }}{y_{\mathrm{label}}^{\prime }}\right)\times 100\%$$

**Figure 4:**
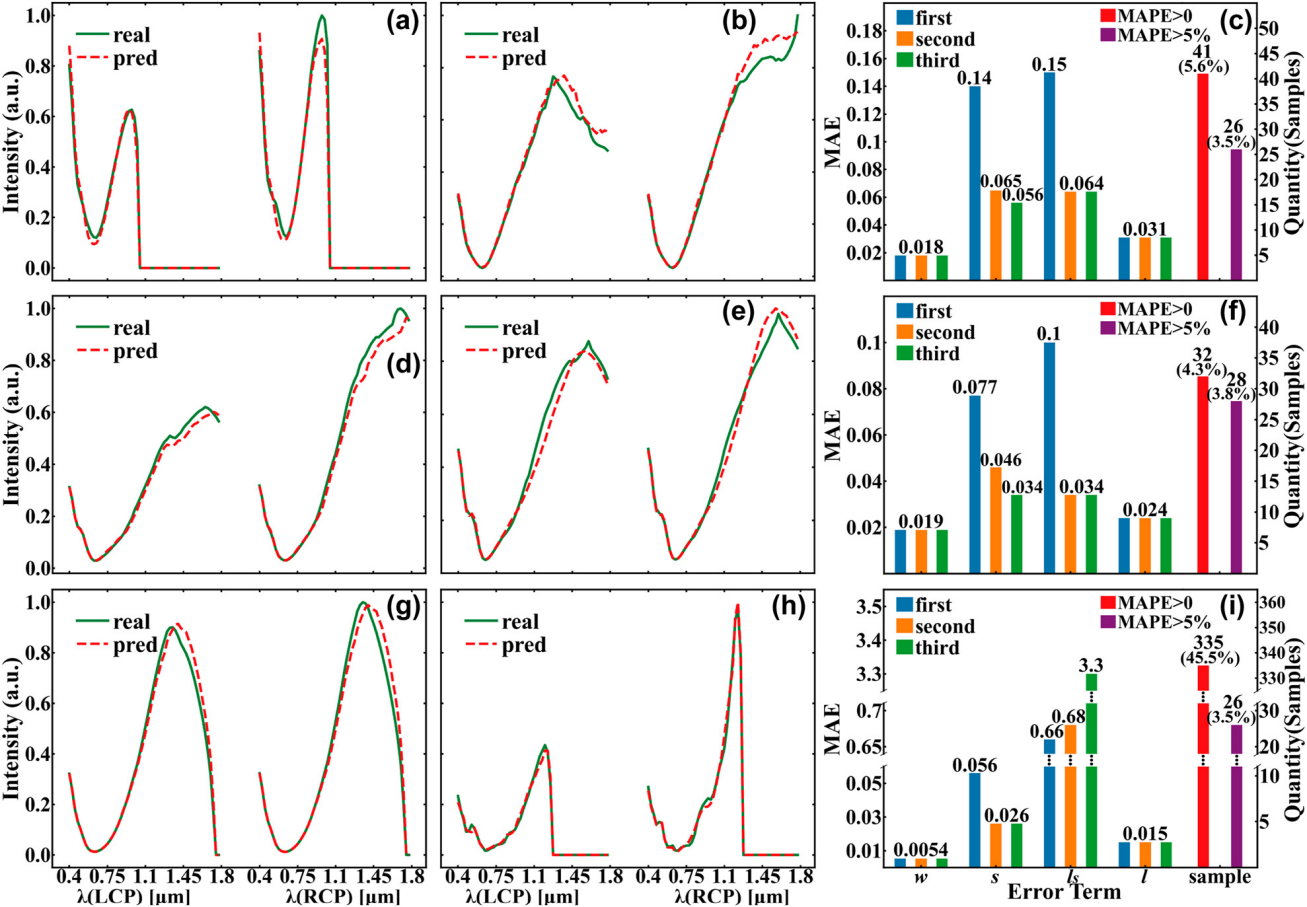
Qualified effects of the inverse design using DEIFS algorithm. (a), (b), (d), (e), (g), (h) Comparison of spectra corresponding to real (green solid lines) and predicted (red dotted lines) geometric parameters for S1, S2 and S3 from top to bottom, respectively. Concretely, their real and predicted parameters and MAPE are shown in Table 2. (c), (f), (i) The errors of the geometric parameters in the iterations and the number and percentage of wrongly predicted samples in the testing for S1, S2, and S3 from top to bottom respectively. Notably, the labels are normalized to be integers so the minimum change in geometric parameters is one rather than the “precision” denoted before.

Only those predictions whose optical response’s error greater than 5% are considered “wrong”. The “right” designs consist of two portions. One is the predictions completely consistent with the real parameters; the other is the samples with tiny errors. The MAPE of the former equals to 0 and that of the latter is in a range of 0–5%. Some predicted samples’ spectra whose MAPE are around 5% are shown in Figure 4(a), (b), (d), (e), (g) and (h), and their corresponding geometric parameters are presented in Table 2. Errors of this magnitude have no significant impact on the results since they hardly alter the spectra, indicating that the rule is reliable in most cases. Additionally, the errors of inverse design covering the entire samples are explicitly displayed in Figure 4 (c), (f) and (i). They distinctly reveal the decreasing errors of parameters in three iterations on the testing dataset containing 737 samples. The two rightmost bars in these three subplots show the number of samples whose predicted and real parameters are not identical. Notably, only the rightmost bar represents the “wrong” designs, and the penultimate bar represents the sum of the quantities of the “wrong” designs and the “right” designs with tiny errors. To highlight the “error term”, the completely correct predictions are not shown in the figure, but this portion accounts for the majority of testing dataset, namely 94.4% for S1, 95.7% for S2, and 54.5% for S3. Obviously, after filtering the samples with tiny errors (i.e., 0 < MAPE ≤5%), the error rates of all three structures are controlled at about 4%. This indicates a relatively low percent of “wrong” designs compared with the “right” ones, illustrating the power of the algorithm in realizing accurate inverse design. In more detail, the four parameters differ in their computational difficulty in terms of parameter errors in the three iterations. Calculating the accurate width and gold length is relatively easy and they are usually selected in the first iteration to assist the design of the remaining two parameters. For S1 and S2, obtaining exact predictions of the space is harder than bridge length while the opposite is true for S3. This phenomenon is basically consistent with their different effects on the spectra, as shown in Table 3. In general, the larger value in the table means the greater influence of each unit on the spectrum when the parameter changes. That is, for these parameters, the algorithm can capture and learn the highly nonlinear relationship between their changes and the corresponding spectra more simply, so the error of their prediction is usually less than the others. There is an exception that the $diff_{l_{s}}$ is greater than *diff*_*l*_ for S3, but its bridge length is too hard to strictly predict for the algorithm, probably due to the multiple solutions introduced by the bridge length. Here, “multiple solutions” means that different combinations of geometric parameters correspond to similar optical responses, which is also difficult to solve in conventional scanning simulations. The DEIFS algorithm tries to minimize its impact which cannot be eliminated due to the underlying physics. In addition, we also utilize the original training dataset only containing the “real” RCWA-derived samples to train the tandem network for comparison, with the results being shown in Tables 4 and 5. It can be easily found that the application of data enhancement can reduce the MAE to no greater than 35% of the original loss in nearly all cases. Therefore, data enhancement can greatly reduce the number of “wrong” designs, revealing the unique benefit of data enhancement. Notably, the errors of results using only the original data are so significant that they are unacceptable for the second and third iterations, so there is no relevant data in Table 5. As for the advantage of iterations, it can be found through comparing the decreasing quantity of “wrong” samples in different iterations. It should be noted that only two test iterations are performed for S3 due to the large prediction error of its bridge length in the third iteration. Thus, in the case that all the results can be well explained, the algorithm is suggested to be very reliable.

**Table 2:** The values of real and predicted parameters and MAPE corresponding to spectra in Figure 4.

Figure	MAPE	Values of geometric parameters							
		*w* (real)	*w* (pred)	*s* (real)	*s* (pred)	*l*_*s*_ (real)	*l*_*s*_ (pred)	*l* (real)	*l* (pred)
4(a)	4.46%	0.175*l*	0.175*l*	0.75*l*	0.95*l*	0.75*l*	0.8*l*	0.9 μm	0.8 μm
4(b)	4.81%	0.3*l*	0.3*l*	0.6*l*	0.55*l*	1.0*l*	1.0*l*	2.0 μm	2.0 μm
4(d)	4.30%	0.125*l*	0.125*l*	0.55*l*	0.55*l*	0.65*l*	0.65*l*	1.9 μm	2.0 μm
4(e)	5.00%	0.15*l*	0.15*l*	0.6*l*	0.7*l*	1.0*l*	0.95*l*	2.0 μm	1.9 μm
4(g)	4.95%	0.1*l*	0.1*l*	1.0*l*	1.0*l*	0.4*l*	0.65*l*	1.3 μm	1.3 μm
4(h)	5.39%	0.125*l*	0.125*l*	0.35*l*	0.35*l*	0.5*l*	0.45*l*	1.4 μm	1.4 μm

**Table 3:** The different effects of the four geometric parameters on the spectra.

Effects	Type of the chiral metamaterials		
	S1	S2	S3
*diff*_*w*_ */diff*_*s*_	1.45	1.51	1.43
*diff*_*s*_ */diff*_*s*_	1.00	1.00	1.00
*diff*_*ls*_ */diff*_*s*_	1.17	1.19	1.30
*diff*_*l*_ */diff*_*s*_	1.26	1.27	1.25

**Table 4:** The different MAE results of the tandem network with and without data enhancement (DE).

Group	MAE of the geometric parameters											
	*w*			*s*			*l*_*s*_			*l*		
	Without DE	With DE	Ratio	Without DE	With DE	Ratio	Without DE	With DE	Ratio	Without DE	With DE	Ratio
S1	0.10	0.018	18%	0.63	0.14	22%	0.50	0.15	30%	0.24	0.031	13%
S2	0.26	0.019	7%	2.4	0.077	3%	1.1	0.10	9%	6.3	0.024	0.4%
S3	0.11	0.0054	5%	0.18	0.056	31%	1.0	0.66	66%	0.043	0.015	35%

**Table 5:** The different sample quantity results of the tandem network with and without DE.

Group	Sample quantities								
	The first iteration			The second iteration			The third iteration		
	MAPE = 0	MAPE >0	MAPE >5%	MAPE = 0	MAPE >0	MAPE >5%	MAPE = 0	MAPE >0	MAPE >5%
S1, without DE	297	440	390						
S1, with DE	583	154	131	690	47	29	696	41	26
S2, without DE	3	734	731						
S2, with DE	634	103	94	699	38	32	705	32	28
S3, without DE	256	481	150						
S3, with DE	382	355	46	402	335	26			

It is worth mentioning that the above results do not take much time compared with the RCWA approach. Traditional RCWA method usually preconfigures a structure and computes its optical response. Then it continuously adjusts the geometric parameters until the spectrum meets the functional requirements. This process is quite time-consuming and difficult to quantify. In contrast, all three iterations of DEIFS algorithm can be completed within 1 s, greatly improving the efficiency of inverse design. According to the experimental results, the algorithm spends 0.75 s achieving the inverse design of 737 samples in the testing dataset.

Besides its high accuracy and ultrafast speed, the flexibility of the algorithm deserves to be mentioned. In this paper, three types of 2D chiral metamaterials are studied, which shows that the algorithm is flexible enough to solve more issues rather than being limited to a specific structure. It is also an advantage of DEIFS algorithm compared with most previous deep learning methods. It is worth mentioning that DEIFS algorithm is probably able to deal with non-unique solution caused by multi-parameters in inverse design via neural network modification, which facilitates better extraction and learning of the potential highly nonlinear relations between the parameters and spectra.

## 5 Further exploration of multi-shaped chiral metamaterials via DEIFS

**Figure 5:**
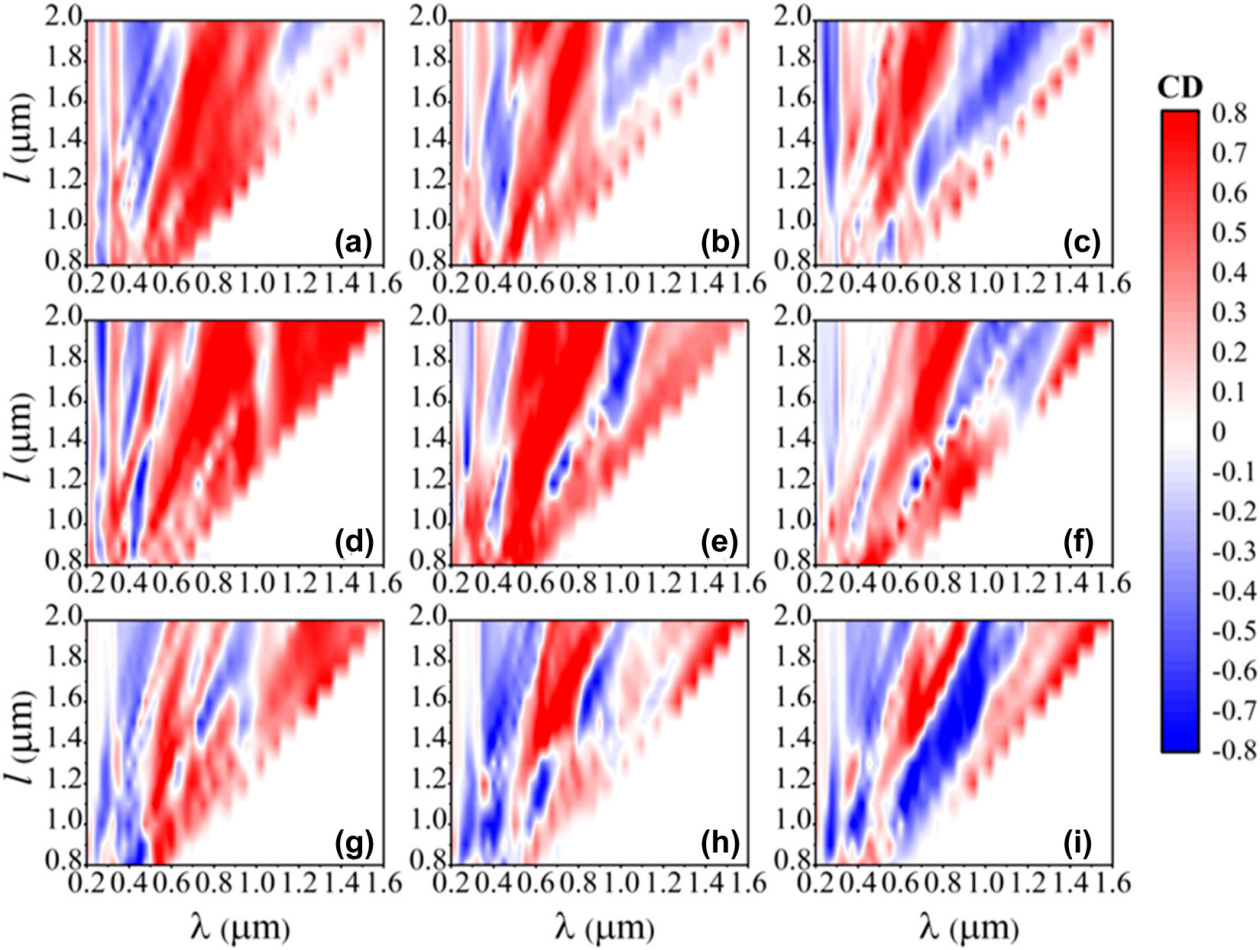
CD contour maps of the third-order diffracted beams in S1–S3 chiral metamaterials, considering various wavelength, bridge length, and gold length. From top to bottom, the panels represent the cases of S1, S2, and S3 metamaterials, respectively. The left to right panels stand for the cases of *l*_*s*_ = 0.4*l*, *l*_*s*_ = 0.6*l*, and *l*_*s*_ = 0.8*l*, with the rest parameters set to *w* = 0.2*l* and *s* = 0.2*l*. All results are calculated utilizing the DEIFS algorithm.

Via DEIFS, we turn to carry out the inverse design for multi-shaped 2D diffractive chiral metamaterials and explore the nonlinear and nonintuitive relationships between chiral parameters and their CD responses. Notably, the shape and unit period are two crucial parameters of 2D metamaterials that dramatically influences chiroptical responses. Normally for a simple grating, its resonant wavelength *λ* and diffraction angle *θ* would obey the grating equation of *a* sin*θ* = *n*·*λ*, with *n* to be the diffraction order. However, this relation is no longer suitable for our case, as the multi-shaped chiral metamaterials cannot be viewed as point sources, whose dimensions are comparatively large for incident wavelength. Accounting for the facts that the unit period satisfies the relation of *a* = 2*l* + 2*s* and the width *w* is proportional to gold length *l*, we begin applying DEIFS algorithm to explore the CD characteristics of third diffraction order lights for S1–S3 metamaterials at divergent gold length *l* and bridge length *l*_*s*_, as shown in Figure 5. The most striking result to emerge from this figure is that the dependence of CD properties on the wavelength λ and gold length *l* is exceptionally nonlinear, for all 3 investigated metamaterials. For most *l* of S1–S3 chiral metamaterials, complex bisignate phenomena are immediately found from CD maps, which are denoted as positive (red) and negative (blue) signs of CD. Take S2 metamaterials for instance (see Figure 5(d)–(f)), when increasing the bridge length *l*_*s*_, bisignate features of CD responses changes significantly, which turns to be more sophisticated at larger *l*_*s*_. Additionally, the blue CD modes play a dominant role across the contour map of S3 metamaterials with *l*_*s*_ = 0.8*l* (see Figure 5(i)), while the red modes turn much stronger in S1 and S2 array with *l*_*s*_ = 0.4*l* (see Figure 5(a) and (d)), with a quasi-balance state between these two modes reached for the remaining chiral modules. This indicates that the variation of bridge length *l*_*s*_ would cause the shape change of chiral metamaterials, and affect the higher-order diffraction modes. In other aspects, the diffraction angles of higher-order diffractive CD responses can be engineered by tuning the unit period, boosting the applications of hypertensive, angle-resolved chirality sensing, and detecting.

**Figure 6:**
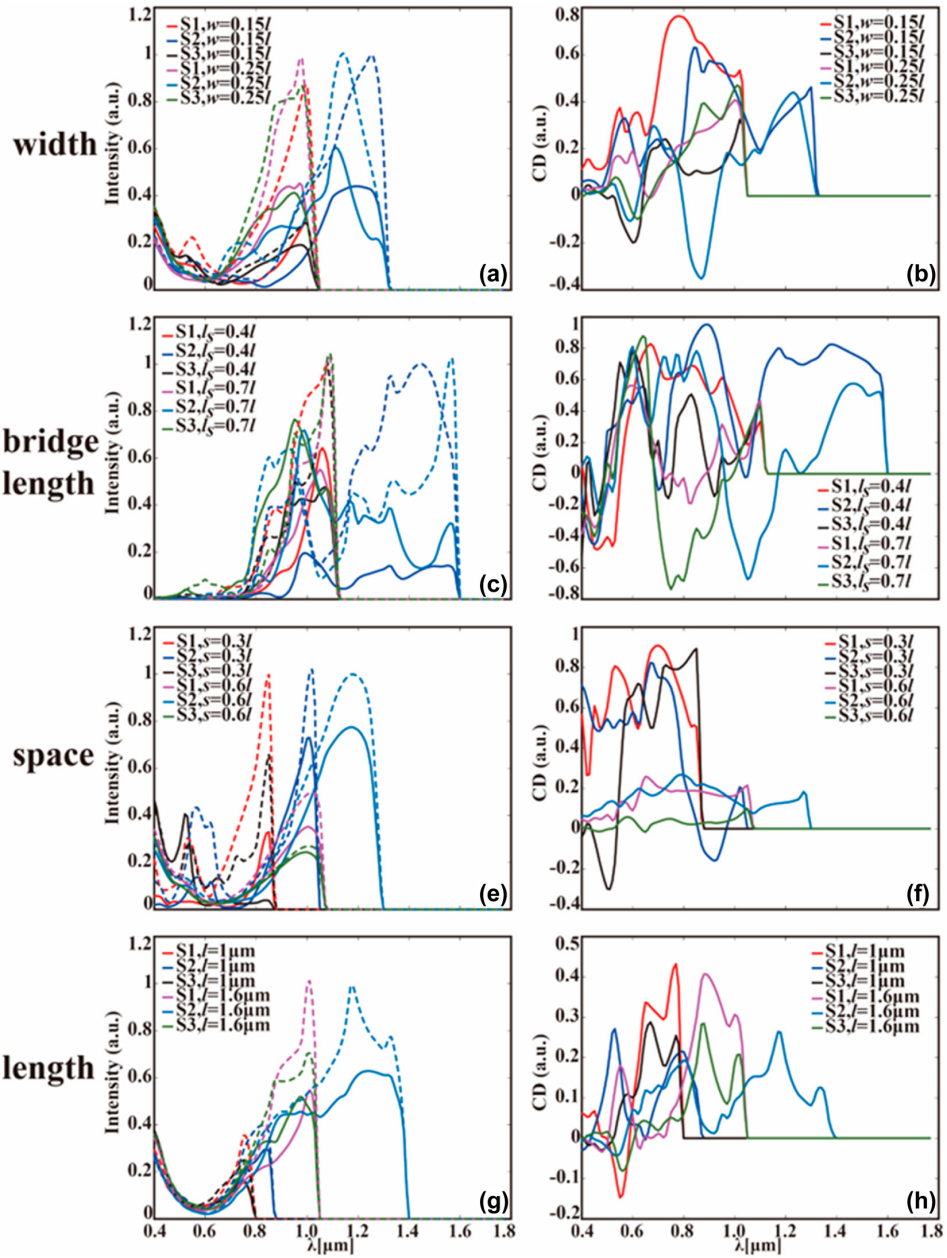
The DEIFS predicted CD spectra of S1–S3 metamaterials possessing four typical geometrical parameters. Precisely, (a)–(d) illustrate the third-order diffraction beam intensity of S1–S3 under irradiation of LCP and RCP light, whereas (e)–(h) demonstrate the corresponding CD responses, with the variables to be width (a) and (e), bridge length (b) and (f), space length (c) and (g), and gold length (d) and (h). It is important to stress that only one key parameter is altered at a time, while the values of rest parameters are fixed: (a) and (e) *s* = 0.4*l*, *l*_*s*_ = 0.5*l*, *l* = 1.4 μm; (b) and (f) *w* = 0.2*l*, s = 0.2*l*, *l* = 2 μm; (c) and (g) *w* = 0.15*l*, *l*_*s*_ = 0.6*l*, *l* = 1.2 μm; (d) and (h) *w* = 0.3*l*, *s* = 0.2*l*, *l*_*s*_ = 0.6*l*. Notably, the solid lines represent the cases of LCP light, whereas the dashed ones stand for the RCP light.

It would be extremely important at this point to study the impact of all typical chiral parameters on the higher-order diffracted chiroptical response of multi-shaped chiral modules. Thus, we illustrate the CD spectra of S1–S3 metamaterials in Figure 6, accounting for the changeable width, bridge length, space length, and gold length, respectively. One remarkable finding from Figure 6(a) is that the LCP/RCP intensity of the third-order diffraction beams is enhanced when gold width increases from *w* = 0.15*l* to *w* = 0.25*l*, but with a slight blue shift of the resonant wavelength, for all S1–S3 metamaterials. However, from Figure 6(d), one can discern that the strongest CD responses of S1 and S2 are observed at *w* = 0.15*l*, while the largest CD of S3 is *w* = 0.25*l*, indicating that the comparably giant LCP and RCP intensity is not a prerequisite to ensure a large CD. When considering the impact of bridge length in details, one can readily discover from Figure 6(b) is that most the LCP and RCP resonant modes locate in the range of 0.8–1.6 μm, whilst the maximum CDs locate in a wider wavelength range (see Figure 6(f)). Another interesting finding from Figure 6(b) and (f) is that increasing bridge length *l*_*s*_ enables the geometric difference for S1–S3 chiral metamaterials, which not only alters the higher-order diffraction modes, but also changes the corresponding chiroptical responses, consistent with results in Figure 6. What follows is the evaluation of the variable of space. It is found from Figure 6 (c) and (g) that both the resonant wavelength of light intensity and CD show redshifts with the increment of space length. In this case, the unit period is enlarged at longer *s* according to *a* = 2*l* + 2*s*, but accompanied by weaker coupling interaction between adjacent modules, ultimately causing variations in CD responses. Lastly, the chiroptical response curves of S1–S3 metamaterials with different gold length and fixed other parameters of *w* = 0.3*l*, *s* = 0.2*l*, and *l*_*s*_ = 0.6*l* are presented in Figure 6 (d) and (h). One significant conclusion is that the resonant wavelength of LCP/RCP light intensity turns larger with an enlarged gold length, and the same is true for the CD resonant wavelength. Furthermore, a highly nonlinear and complicated relation between the CD response and gold length or unit period is also discovered, in agreement with Figure 6.

## 6 Conclusions

In summary, a DEIFS algorithm based on DATA AUGMENTATION and REGRESSOR CHAINS had been developed. Through DEIFS, inverse design for multiple 2D chiral metamaterials was accomplished in a significantly-fast and extremely-accurate way. Firstly, the RCWA method was applied to provide an original dataset containing the optical circular dichroism responses of three chiral metamaterials (S1, S2, and S3), with the forward network generating supplementary training data. This greatly reduces the dependence on simulations in the inverse design process of programmable metamaterials. And then, the enhanced dataset was utilized to iteratively complete the inverse design step by step, avoiding the conventional time-consuming iterative process. The inverse-design results can be well explained by the DEIFS algorithm in perspective of data, indicating its high dependability. Equally important, using the DEIFS network, the complex and nonlinear dispersion relationships between CD responses and shape, unit period, width, bridge length, and separation length of diffractive chiral metamaterials had also been addressed. The flexibility and accuracy of the DEIFS algorithm are extremely excellent which demonstrates its broad prospects in inverse design of optical devices. This work represents a giant progress in the aspect of multi-task inverse design of 2D chiral metamaterials and promotes the excellent potentials of DEIFS in applications of optical chirality related nano-devices and complex but programmable metamaterials for optical coding and information processing.
